# Serine substitutions are linked to codon usage and differ for variable and conserved protein regions

**DOI:** 10.1038/s41598-019-53452-3

**Published:** 2019-11-21

**Authors:** Gregory W. Schwartz, Tair Shauli, Michal Linial, Uri Hershberg

**Affiliations:** 1 Department of Pathology and Laboratory Medicine, Perelman School of Medicine at the University of Pennsylvania, Philadelphia, USA; 2 School of Computer Science and Engineering, The Hebrew University of Jerusalem, Jerusalem, Israel; 3 Department of Biological Chemistry, Institute of Life Sciences, The Hebrew University of Jerusalem, Jerusalem, Israel; 4 Drexel School of Biomedical Engineering, Science and Health Systems, Drexel University, Philadelphia, USA; 5 Department of Microbiology and Immunology, Drexel College of Medicine, Drexel University, Philadelphia, USA; 6 Department of Human Biology, Faculty of Science, University of Haifa, Haifa, Israel

**Keywords:** Genome informatics, Molecular evolution, Complexity, Evolvability

## Abstract

Serine is the only amino acid that is encoded by two disjoint codon sets (TCN & AGY) so that a tandem substitution of two nucleotides is required to switch between the two sets. We show that these codon sets underlie distinct substitution patterns at positions subject to purifying and diversifying selections. We found that in humans, positions that are conserved among ~100 vertebrates, and thus subjected to purifying selection, are enriched for substitutions involving serine (TCN, denoted S′), proline, and alanine, (S′PA). In contrast, the less conserved positions are enriched for serine encoded with AGY codons (denoted S″), glycine and asparagine, (GS″N). We tested this phenomenon in the HIV envelope glycoprotein (gp120), and the V-gene that encodes B-cell receptors/antibodies. These fast evolving proteins both have hypervariable positions, which are under diversifying selection, closely adjacent to highly conserved structural regions. In both instances, we identified an opposite abundance of two groups of serine substitutions, with enrichment of S′PA in the conserved positions, and GS″N in the hypervariable regions. Finally, we analyzed the substitutions across 60,000 individual human exomes to show that, when serine has a specific functional constraint of phosphorylation capability, S′ codons are 32-folds less prone than S″ to substitutions to Threonine or Tyrosine that could potentially retain the phosphorylation site capacity. Combined, our results, that cover evolutionary signals at different temporal scales, demonstrate that through its encoding by two codon sets, serine allows for the existence of alternating substitution patterns within positions of functional maintenance versus sites of rapid diversification.

## Introduction

**Figure 1:**
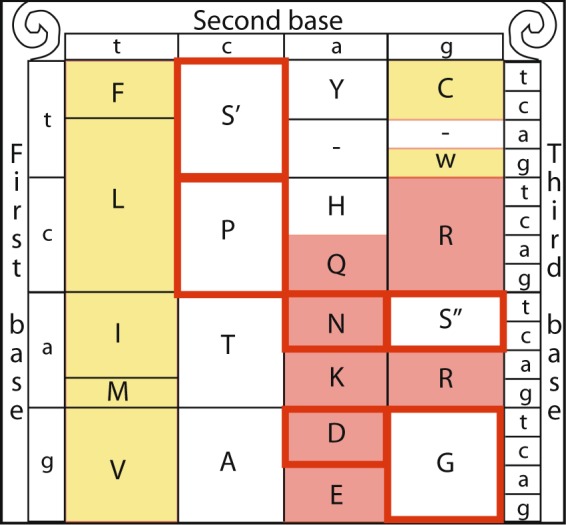
The genetic code annotated for 4 types of amino acid characteristics. Hydrophobic (yellow fill), Neutral hydrophobicity (white), Hydrophilic (red) and β−Turn (red frame)^3,6,7^.

Due to the redundancy of the genetic code most amino acids are encoded by more than one codon. In most cases the codons encoding for a specific amino acid differ only by a single nucleotide (typically the third one). However, serine is unique among the 20 amino acids in that it is encoded by two disjoint sets of codons that require a tandem substitution of two nucleotides to switch between the two sets, one comprised of the codons TCT, TCC, TCA and TCG (TCN, denoted set S′) and the other set includes only two codons - AGT and AGC (AGY, denoted set S′′)^1^. The two sets of codons encoding serine cannot be connected through a single point mutation^1^. The serine’s codon structure allows it to participate in two separate substitution patterns, each of which conserves different amino acid characteristics. The S′′ single mutation transition substitution category includes the amino acids serine, in addition to transition neighbors^2–4^ glycine (G), and asparagine (N). In contrast, the S′ single mutation transition substitution category includes serine, proline (P), and alanine (A)^3,5,6^ (Fig. 1). The substitution of S′ to amino acids P and A occur by a single mutation at the first codon position. The substitution from serine to threonine (T) is not included either of the substitution sets, as it can be a result of a single nucleotide change from either S′ of S″. While the amino acid substitution derived from S′ are enriched in conserved β-turns^3,6,7^, those derived from S″ are generally of a neutral hydrophobicity (i.e., amino acids that are neither strongly hydrophobic or hydrophilic)^3,6^.

We tested the usage of the two patterns of serine substitutions in different evolutionary contexts. Substitution patterns were studied across 99 vertebrates in HIV envelope glycoprotein, and in humans for the germline and somatic diversification of the immune B cell receptor repertoire. We demonstrated that the special form of the codon redundancy encoding serine underlies serine’s participation in two distinct substitution patterns. One of which (amongst the amino acids – GS″N) is more abundant in sequence positions subject to positive, diversifying selection, while the other (amongst - S′PA) is more abundant at highly constrained positions, subject to strong purifying selection. Additionally, we detected that substitutions of the S′ set associated with phosphorylation sites that are under a stronger constraint relative to that of the S″ set. It therefore appears that the genetic code has evolved to specifically allow serine to participate in two types of selective regimes, one that complies with strong constraint and another separated regime for diversification.

## Results

### Serine displays a natural partition to two codon sets with distinct substitution patterns

**Figure 2:**
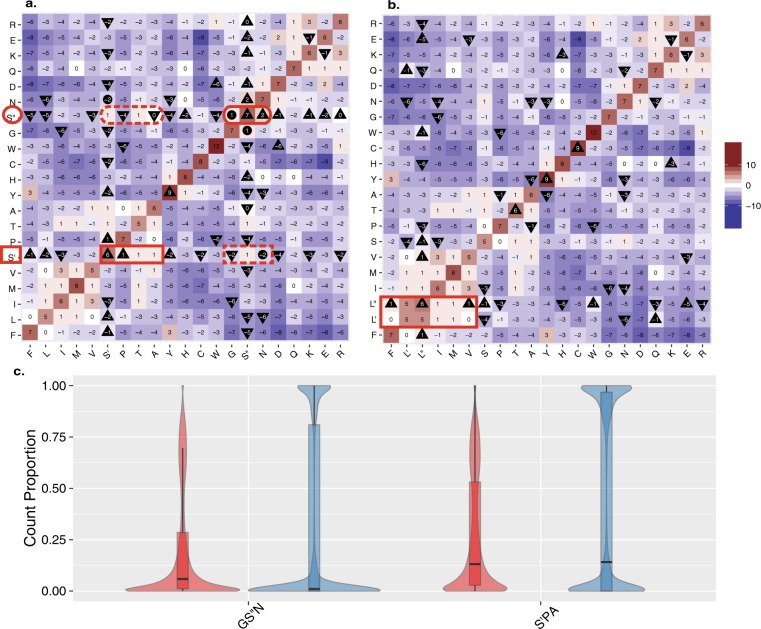
Comparison of codon and amino acid substitutions in the alignment database of 99 vertebrates species. (**a**) BLOSUM heat map from the UCSC alignments generated while considering substitutions to/from S′ and S′′ separately. Heat map colored from blue (<−10) to red (>10) with a midpoint gradient of white (0). A triangle pointing up designates an increase in value compared to the unmanipulated UCSC based BLOSUM (Sup. Fig. 1), while a triangle pointing down represents a decrease in value. A circle signifies a sign flip (+to - or vice versa) compared to the unmanipulated BLOSUM (Sup. Fig. 1). Note that the viable/positive substitutions from S′′ (*red oval*) are all negative from S′ (*red rectangle broken line*), similarly viable/positive substitutions from S′ (*red rectangle*) are not from S′′(*red oval broken line*^).^ T, which is equally reachable from both S′ and S″ remains unchanged. (**b**) The BLOSUM generated by considering substitutions to/from L′ and L′′ separately ^(^details as in *a*). Note how no positive substitutions from L′ or L′′ are negative from the other and all negative substitutions are shared (positive marked in red rectangle). (**c**) Considering only those positions where serine is meaningfully present (see Methods), we show the fraction of species sequence positions that are GS′′N *(two plots on the left)* or S′PA *(two plots on the right)*. For each set of amino acids, the species sequence positions are split by the measure of diversity into positions above the upper quartile of diversity (*red*) and below the lower quartile of diversity (*blue*). Each distribution is represented by a box plot overlaying a violin plot. The black line represents the median value of each group.

We hypothesized that each of the individual serine codon sets had its own preferred substitutions, and that these two groups of serine substitutions would inhabit different position and properties in protein sequence. To characterize the impact of the differential distribution of serine on its genome wide substitution patterns, we reconstructed a new version of the well characterized BLOcks SUbstitution Matrix (BLOSUM)^8^ (see Methods) that split the substitution matrix for serine into S′ and S′′. We found that the two codon-based serine substitution sets differ in the direction of their substitution scores. The substitution values from S′′ to G and N are both positive, indicating they occur more frequently than expected by chance, while substitution values from S′ to G and N are negative. At the same time, substitution values from S′ to P and A are positive while those from S′′ are not (Fig. 2a).

In accordance with the original BLOSUM, in our reconstructed matrix, synonymous mutations are always associated with positive values. However, we find that synonymous mutations of S′ or S′′ (i.e. the values on the diagonal) all have values approximately six-fold higher than the value reported for the synonymous mutation in which S′ is substituted to S′′ or vice versa. The overlooked mutation distance between the synonymous mutations from S′ to S′′, is not considered by the classical BLOSUM. Remarkably, in all cases when we consider the differences between S′ and S′′ substitutions with respect to the substitutions of serine in the original, unmodified version of the BLOSUM matrix, we find that they have also changed appropriately. Moreover, in the case of substitutions from S′′, several types of substitutions flipped the sign of their values from negative to positive compared to the original BLOSUM (Fig. 2a, Sup. Fig. S1a).

In addition to serine, leucine (L) and arginine (R) are also encoded by 6 codons which are composed of two sets. To check whether the differences of our S′/S′′ matrix from the standard BLOSUM could be merely attributed to a more detailed description of codons, we repeated the analysis and tested the natural split of leucine into CTN (CTT, CTC, CTA, CTG, denoted L′ set) and TTR (TTA, TTG, denoted L″ set). For L′ and L″ we found no difference in the sign of the substitution scores. The leucine substitution scores change in their extent but not in their tendency for being substituted (as reflected by the sign of the substitution values). Furthermore, the synonymous changes of L′ to L′, L′′ to L′′, and the cross sets of L′ to L′′ and L′′ to L′ all receive approximately equal values (Fig. 2b). We observed the same trend when arginine was tested. Splitting arginine into R′ and R′′ (Sup. Fig. 1b) was not associated with any natural partition in the newly reconstructed substitution matrix. It is important to note that splitting either serine, arginine or leucine into two substitution groups, has a negligible effect on the substitution patterns of all other amino acids (Sup. Fig. S1).

In this way we find that considering serine codon usage divides serine into two substitution groups. Based on the different properties of the serine substitutions groups, we postulated that the neutral amino acids that have a propensity towards β-turns (GS′′N)^7^ would be preferred at positions where the diversity of permissible amino acid usage is high, while the generally neutral amino acids (S′PA) would be prevalent in more conserved positions. The underlying rationale stems from the dominant role of β-turns in contact regions of receptors. Exchange among amino acids that share a propensity to form β-turn is most likely to modify contact parameters while preserving the potential of the region to engage in binding and protein-protein interaction.

### The distinct patterns of serine substitutions correlate with amino acid sequence diversity

To test the second part of our hypothesis, that the two distinct serine substitution patterns will be differentially expressed in conserved and diversifying amino acid positions, we assessed the correlation between the amino acid diversity and the fraction of amino acids occupied by the two substitution serine groups: GS′′N and S′PA. The analysis covered all positions in the human proteome where serine was ″meaningfully″ present within multiple alignments of 99 vertebrate genomes (represented in UCSC Genome Browser Database^9^, see Methods). Using the measure of diversity^10^, we considered serine to be meaningful at a position if it was abundant enough to contribute to the diversity of the sample, at that position^10^ (see Methods). Focusing at positions in which serine (S′ and/or S′′) was a meaningful amino acid^10^ (see Methods) we found that the fraction of GS′′N usage at each sequence position showed a weak but significant positive correlation to diversity of amino acids at that position while the fraction of S′PA usage showed a negative correlation (Spearman’s rank correlation for GS″N rho = 0.146, p-value < 0.001, and for S′PA rho = −0.126, p value < 0.001). Inspecting more explicitly the top and bottom quartiles of the diversity distributions at different positions, we found the skew in the substitution patterns to be even more pronounced. We compared the levels of GS′′N and S′PA in the top and bottom quartiles of positions ranked according to their degree of diversity. We found that the fraction of GS′′N in most diverse positions was 6 folds more prevalent compared to the least diverse positions (median fraction GS′′N = 0.0588 vs. 0.0103). Based on the same analysis, the fraction of S′PA shows the opposite trend, albeit to a lesser extent (median fraction S′PA = 0.131 vs. 0.141). In both cases these differences are significant (Mann-Whitney U test: GS′′N *p value < 0.001*, S′PA *p value < 0.001*) (Fig. 2c).

### GS′′N is over utilized in the fast diversifying regions of HIV envelope glycoprotein gp120

**Figure 3:**
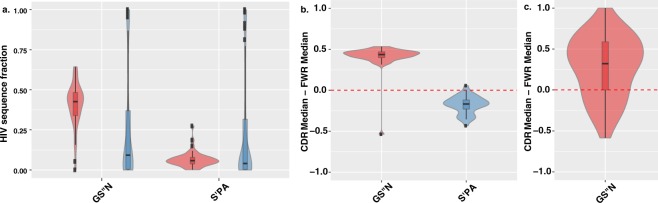
GS′′N and S′PA biases between regions of differing diversity. (**a**) Fraction of GS′′N (*left*) or S′PA (*right*) amino acids in HIV diverse hotspot regions (*red*) vs. other more conserved positions (*blue*). The distributions are represented by a box plot overlaying a violin plot. (**b**) Looking across clone populations in 40 humans we show (i) the distribution of the difference per individual of the median fraction of clones that have more GS′′N (out total amino acids) in CDR compared to FWR germline positions and the median fraction of clones that have more GS′′N in FWR compared to CDR positions, where serine is meaningful (*red*); (ii) the distribution of the difference per individual of the median fraction of clones that have more S′PA (out total amino acids) in CDR compared to FWR germline positions and the median fraction of clones that have more S′PA in FWR compared to CDR positions, where serine is meaningful (*blue*). (**c**) Distribution across individuals of the difference between median fraction of clones that have a higher fraction of GS′′N (out of GS′′N + S′PT) at a CDR somatic substitution position and the median fraction of clones that have a higher fraction of GS′′N (out of GS′′N + S′PT) at FWR somatic substitution positions.

We next tested the possibility that the partition of S′ and S′′ was not only associated with conservation at the level of long-range evolution taxonomy but also associated with high diversity positions that govern protein interaction. We tested the HIV envelope gp120, an exposed glycoprotein on the surface of the HIV envelope which is essential to virus entry into cells. The protein gp120 is a key determinant in the virus’ ability to bind surface receptors, including CD4, that lead to viral fusion^11,12^. It is therefore a likely subject for strong evolutionary constraints. At the same time, gp120 is a prominent antigen that undergoes rapid adaptation to evade the immune system^13,14^. It is thus an ideal showcase of a protein sequence comprised of amino acid positions that are highly conserved and others that are hypervariable. Comparing the substitution patterns in these two types of positions, we considered only positions in which serine (S′ and/or S′′) was a meaningful amino acid (see Methods). The analysis was performed on a dataset of 4173 gp120 sequences^2^ (see Methods). Similarly, to the observations from the data set of vertebrate conservation (Fig. 2c**)**, we found that GS′′N is over utilized in the fast diversifying regions of gp120^2–4^ (referred to as hotspot, median = 0.426), compared to the rest of the protein (median = 0.09) (Mann-Whitney U test: *p value = 6.11 × 10*^*−6*^). At the same time, S′PA shows a significant decrease in representation between hotspots (median = 0.06) and the rest of the protein (median = 0.152) (*p value* =* 0.04*). (Fig. 3a and Sup. Fig. S2).

### A selection footprint in both germline and somatic B cell receptor populations

As our final example of the different substitution patterns of serine in diversifying and conserved positions, we considered both the somatic and germline substitution patterns in B cell receptors. Let us first give a short introduction of this system to state the foundation for B cell receptors as a model of evolutionary selection process, emphasizing the special nature of somatic B cell receptor diversification. The somatic selection process is especially informative as we can elucidate its germline source and thus track not only substitution patterns but also, and uniquely, their amino acid source. B cells undergo two stages of differentiation and selection of their B cell receptors. Both stages are essential for the creation of a varied immune repertoire to recognize and fight diseases. In the first stage, B cells located in the bone marrow recombine germline V(D) and J gene segments^3,5,6^ to create heavy and light chains which, when joined, form a unique B cell receptor^3,6,7,15^. B cells that form a functional receptor in this fashion are selected and proliferate, producing B cell clones, each with a common progenitor and a common receptor type. During the immune response, the naive B cell population undergoes an additional process of affinity maturation. B cells proliferate, rapidly mutate their B cell receptor genes and die, such that the B cells with B cell receptors of higher binding to the antigens of the disease dominate the B cell population. Since most of the human V, D, and J germline gene segments that recombine to encode the B cell receptor are known^7,16^, we are able to assign every observed mutant B cell receptor sequence to its germline origin. In this way, we can identify clonotypes of sets of mutant B cells with common progenitors and characterize the precise types of surviving substitutions along with their amino acid sources. Moreover, we can determine for every amino acid in a sequence if it is germline encoded or the result of a somatic substitution during an immune response. Finally, the B cell receptor, much like the HIV gp120, is also divided into highly variable complementarity determining regions (CDRs) and more constrained framework regions (FWRs)^9,17^. The CDRs interact with antigens and therefore need to rapidly adapt and diversify and are thus under diversifying selection^10,18–20^. On the other hand, the FWRs serve as the backbones of the receptor. As such, these regions are constrained to maintain the rigidly of their structure, and thus are mostly under purifying selection^10,18,21^.

### The two serine substitution patterns differ in their prevalence in the variable and conserved positions of the B cell receptor repertoire

We analyzed the B cell repertoires of 40 human individuals from three different geographical location across the globe^10,22–24^ (see Methods). For each individual we characterized the full set of heavy chain sequences belonging to the B cell receptor populations. The repertoires of sequences were divided into clones, as described above (and see Methods). Focusing on the V gene segment of the B cell receptor, whose germline is clearly defined, we annotated the unique positions in each clone which had either undergone an amino acid substitution or remained encoded for their germline amino acids. We only considered the positions in which serine (S′ or S′′) was a meaningful amino acid based on the diversity of the germline positions (see Methods). Such positions were divided according to their positions into those found in the hypervariable CDRs and the conserved FWRs^8,20,25^.

For each position we counted the number of clones in an individual in which it was germline encoded and either GS′′N or S′PA. We then calculated the median level of GS′′N and S′PA usage in each region (CDR or FWR) across the serine positions. To assess whether the CDR utilized more GS′′N amino acids with respect to FWR, we performed a Wilcoxon signed-rank tests across all 40 individuals comparing individual median level of the deviation of GS′′N in both B cell receptor regions (i.e. CDR minus FWR). We then repeated the same test on S′PA levels across all 40 individuals. When considering germline positions, we found quite clearly and significantly that GS′′N was over abundant in the CDR of individuals while S′PA was over abundant in the FWR (Wilcoxon signed-rank test shows successes for GS′′N at 39 out of 40, *p* = *7.91 × 10*^*−9*^ and S′PA in only 1 out of 40, *p = 9.09 × 10*^*−12*^) (Fig. 3b and Sup. Fig. 2).

### Somatic mutation and selection processes is skewed towards high abundance of GS″N in the highly variable CDR regions

Having established that germline positions of the B cell receptor genes exhibit a preference for GS′′N in the hypervariable (CDRs) regions and S′PA in the more conserved ones (FWRs), we now asked whether such bias also revealed itself for the somatic mutations that signify substitutions with shorter timescale and high level of mutations. To this end, we compared GS′′N somatic substitutions out of the total GS′′N + S′PA substitutions, for each individual with success being when the fraction of GS′′N CDR to the fraction GS′′N FWR is positive (i.e.>0). This test is based on the notion that as FWR regions by and large do not show substitutions. We found that in the somatic substitution as well, the same pattern holds. Specifically, GS′′N is over abundant in the CDR compared to FWR (Wilcoxon signed-rank test reported a success for 27 out of 39, *p* = *6.52* × *10*^*−5*^) (Fig. 3c and Sup. Fig. 4).

### S″ phosphorylation sites are more prone to substitutions relative to those encoded by S′

**Figure 4:**
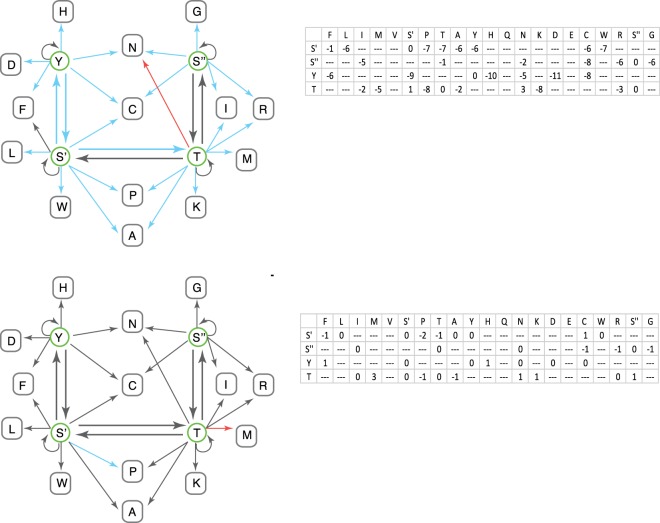
Serine phosphorylation sites show differences in conservation depending on codon usage. Substitution network based on single point mutations based on the aggregation of human population polymorphism from the ExAC database. The tendency of substitution for S, T and Y for all phosphorylated positions *(Top)*. All S, T and Y sites in the human proteome *(Bottom)*. Arrows indicate the substitution directionality. The color of the arrow captures the relative abundance of the substitutions compared to the expected patterns of mutation as calculated directly from the mutations in the third codon positions of 4-fold degenerate amino acids. Substitutions less abundant than expected with a ratio < −1 *(blue)***;** substitutions more abundant than expected with a ratio > 1 *(red)* and substitutions that are at a similar degree as expected (i.e., −1 < ratio < 1) (gray). Values are rounded to show the log power of the substitution abundance. The exact range of the relative abundance of all substitutions from S, Y and T to any other amino acid is shown next to the network view.

To directly show a possible functional consequence to the use of S′ or S″ and their permissible substitutions, we focused on the unique capacity of a subset of serine residues to be modified by serine/threonine kinases. We hypothesized that the tendency of phosphorserine substitution will be biased according to the type of serine encoding sets^26^. In all eukaryotes, serine (S), threonine (T) and tyrosine (Y) are the only amino acids that can be modified by kinases. To test whether there is a difference in the substitution patterns of S′ and S″ in the context of phosphorylation sites, we considered the coding variations from the human Exome Aggregation Consortium (ExAC) dataset. The ExAC dataset aggregates exome polymorphic sites of 60,706 unrelated healthy individuals. Altogether about 8.3 million variations across the human population are reported, many occur at extremely low frequency (i.e. reported for a single individual). We mapped on the ExAC coding exomes 37,565 experimentally observed phosphorylated sites, among them 30,219 are phosphoserine (see Methods). We reanalyzed the ExAC data and quantified the tendency of phosphoserine (p-S) to exhibit substitutions that could maintain its phosphorylation capacity when encoded by S′ or by S″ codon sets. To this end, we first built a substitution model that is based on the neutral model from all reported ExAC variations affecting the third position of the 4-fold degenerate amino acid codons (covers valine, proline, threonine, alanine and glycine, see Methods and Sup. Fig. 5). Looking across all phosphorylation sites, we compared the observed substitutions following single point mutations to the expected pattern of the 4-fold degenerate model. As seen in Fig. 4, a single point mutation from S′ and S″ would lead only to cysteine and threonine. Evidently, a change to cysteine would necessarily leads to a loss of a phosphosite, and this is equally selected against from both S′ and S″ when compared to the l mutation model based on the 4-fold degenerate amino acids. However, we found a strong bias in the substitutions to threonine (T) from S′ or S″. Notably, substitution from serine to threonine is likely to maintain the phosphorylation potential due to the overlap in S/T kinases specificity^27^. While the S′ to T substitution is negatively selected against compared to the 4-fold degenerate mutation model, substitutions from S″ to T are not. The S′ codons are 32 folds less prone to alterations that retain phosphorylation capacity relative to S″ (Fig. 4). This result is in accord with our observation for the non-randomized alternative positioning of S′ and S″ in the genome. The bias against S′ to T substitutions is not symmetrical^28^, we found that the threonine phosphorylation (p-T) sites have apparently an equal frequency of substitutions to both S′ and S″ (Fig. 4, top). When we extended our analysis to all single point mutations in any appearance of S, T and Y across the ExAC, we found that in contrast to our observation regarding the unique bias of functional p-S sites, the substitutions patterns of single point mutations could be primarily explained by the 4-fold degenerate model of mutations (Fig. 4, bottom).

Our results regarding p-S substantiate the notion of a differential selection pressures which are associated with serine encoded by S′ or S″ codon sets. In the case of p-S, we observed that S′ sites are indicative by a reduced tendency to be altered relative to S″, which is in accord with a S′ being engaged in a more conservative substitution patterns when compared to S″.

## Discussion

In the results above we have characterized patterns of substitution of serine both at the whole genome level under long-range evolution comparing the human genome to 99 other species, and at the human population level, analyzing protein substitutions from over 60,000 healthy individuals. We further tested our hypothesis by focusing on two specific biological contexts, the somatic changes for B-cell receptors and gp120 an envelop gene of HIV. In both contexts, the genes include in single polypeptide chain distinguishable regions that are subjected to diversifying (positive) selection and other regions subjected to strong evolutionary constraints (purifying selection). Across all our examined datasets, we found a clear segregation of amino acid substitutions that are predicted by the division of serine encoding according to the genetic code. We show that the bias in serine codon usage previously found in B cell receptor repertoires^29^ has a role in maintaining diversity beyond the immune B cell receptor repertoire. Indeed, it underlies a more general segregation in amino acid substitution patterns that divides serine substitution into two groups linked to the diversity and functionality of gene products. The first group (GS″N), mostly conserve for β-turns, are found in protein regions subject to diversifying selection (e.g., protein contact regions). In contrast, the second set (S′PA) comprise of more generally neutral amino acids and are found in conserved protein regions, subject to stronger evolutionary constraints. To show that S′ is under stricter purifying selection from a more functional perspective we looked also at the substitution patterns of p-S sites in the human population (from ExAC dataset). We showed that while the majority of the phosphorylation sites in the human proteome are p-S (80.4%) of which ~60% are encoded by the S′ codon set. Still, across all p-S sites we found that substitutions from S′ showed a substantial negative selection to threonine, while no such selection is observed for serine that are encoded by the S″ codon set (Fig. 4).

## Conclusion

We have thus shown that in biological selection processes the codons of serine indicate different types of selection for the amino acid and its permissible substitutions. We have shown the importance of this special characteristic of serine, in general and for phosphorylation sites, across multiple scales of evolutionary selection: across species, within human population and for the somatic B cell selection and viral quasi species. At all these scales of selection the S′ codon set is under a stronger purifying selection while S″ codon set tends to undergo diversifying selection, as is reflected from protein sequence, structure and function.

Based on the cumulative observations from vertebrates and human-centric evolution, immune and viral selection we find that in highly diversified positions of amino acids, when serine is present, it is more often encoded by AGY and will substitute in addition to any synonymous changes to glycine and asparagine (GS″N). In contrast, in highly conserved positions serine is more often encoded by TCN and will tend to substitute in a non-synonymous form to proline and alanine (S′PA). In this way we show that codon usage and not just amino acid type serves as an indicator of selection. We provide a further support for the view that the genetic code has evolved to allow maintenance of several types of substitution patterns.

## Materials and Methods

### Analysis of whole genome exon sequences

#### Sequence collection

All 162,633 known canonical exon gene sequences of all isoforms from the multiple alignments of 99 vertebrate genomes with human dataset were obtained from the UCSC Genome Browser database^9,13,14^. A list of each vertebrate used by UCSC, along with their methodology and parameters, can be found at http://hgdownload.soe.ucsc.edu/goldenPath/hg38/multiz100way/.

#### BLOSUM analysis

BLOSUMs were created using our own implementation of the BLOSUM algorithm^2,8^ in order to account for treating S′/S′′, L′/L′′ and R′/R″ differently. The code is open source and available at https://github.com/GregorySchwartz/blosum

#### Calculation of by position diversity of amino acids

For each gene isoform in the data set, the diversity of amino acids was calculated at each position. In order to avoid bias of highly abundant or rare amino acids, the measurements were taken with an order of one^30^. The calculated per position diversity was used to estimate the meaningful types of amino acids found at each position. At each position, only the *n* most abundant types of amino acids were included, where *n* is the amino acid diversity at that position. We defined meaningfully present serine positions as those in which serine was one of its *n* most abundant amino acids^10,19^. Positions were gathered into four groups based on the quartiles of diversity, with the high diversity positions having diversities greater than the upper quartile and low diversity positions having diversities less than the lower quartile, excluding outliers.

#### GS′′N and S′PA comparisons

Within the amino acid positions where serine was a meaningful amino acid, the abundance of GS′′N and S′PA, normalized by the number of sequences at each position across all sequences (the ″fraction″ of each category at a position), were compared in the high diversity and low diversity positions. Spearman’s rank correlation was used to calculate the correlation of GS′′N or S′PT fractions with diversity. All comparisons between the upper and lower diversity positions and between GS′′N and S′PA were done using the Mann-Whitney U two-tailed test.

### Analysis of gp120

We collected 4173 HIV-1 env gene sequences filtered by alignment to HXB2 HIV reference genome from the Los Alamos National Laboratory HIV Database (http://www.hiv.lanl.gov/)^2^ and analyzed their gp120 region. We considered all 145 positions in which S (S′ and/or S′′) was a meaningful amino acid, defined as in the known exon analysis above. Within these amino acid positions, the abundance of GS′′N and S′PA was normalized by the number of sequences at each position across all 4173 sequences. We then compared the abundance of the two substitution groups in the 47 hypervariable positions^2^ and in the 98 remaining positions (Sup. Fig. S3). Following the above results significance of all comparisons was calculated using the Mann-Whitney U one-tailed test.

### Analysis of the heavy chain B cell receptor

#### Sequence collection

Heavy chain B cell receptor sequences were gathered from four different groups of individuals in different populations: (1) Peripheral blood samples from 3 young healthy individuals (D1)^22^; (2) 6 young (D2 young) and 6 old (D2 old) patients sampled at immunization to influenza and 7 or 28 days after^23^; (3) 14 Papua New Guinea individual repertoires (D3)^24^, and (4) 12 Australian individual repertoires (D4)^24^. Samples D1 and D2 were sequenced using the Roche 454 sequencing technology (FLX Titanium)^22,23^, while D3 and D4 where Sanger sequenced on an Applied Biosystems (ABI 3730) machine^24^. Since individuals and samples were compared, individuals that had < 3 sequences were removed. Therefore, D3 01 in the D3 data set^24^ was removed.

#### Clonal definition and mutation counts

In all cases, all sequences were locally aligned using Smith-Waterman with a gap penalty of 16 to conform to the ImMunoGeneTics database (IMGT) definitions and position numbering^25^. To these we added aboriginal germlines to account for alleles evolved in New Guinea^31^. Only the V gene part of the gene was analysed (to amino acid position 106). The CDR and FWR were modified IMGT definitions that were used in previous analysis: FWR1 = 1–24; CDR1 = 25–40; FWR2 = 41–53; CDR2 = 56–65; FWR3 = 66–104; CDR3 = 105–1063^20,25^. To avoid possible ambiguous germline V gene assignments, sequences were removed if they were with over 30% of their nucleotides being mutated. This filtering resulted in 1336 sequences from D1, 20,102 sequences from D2, 1098 sequences D3, and 636 sequences from D4, resulting in data from 40 individuals.

The sequences were separated into clones identified by having the same V gene, J gene, and CDR3 length. This resulted in 1,290 clones from D1, 9,391 clones from D2, 699 clones from D3, and 231 clones D4. The exact number of positions sequences in the different data sets varies. Therefore, in order to have each amino acid sequence position represented in most if not all clones, positions represented in less than 30 sequences in any of the data sets were excluded. Thus all V genes were analyzed only in the following IMGT positions: 25–30, 35–59, 63–72, 74–10625 Within each clone, each mutational event was counted only once. A mutational event in the recombined data sets was defined as one nucleotide change in a codon.

#### Calculation of by position diversity of amino acids

For each individual in each data set, we separately calculated the diversity of germline amino acids and the diversity of amino acids substituted from their respective germline at each position across clones.

#### GS′′N and S′PA comparisons

GS′′N and/or S′PA levels amongst somatically substituted and germline maintained amino acids in each position in each individual, were compared only in the 27 positions in which S (S′ and/or S′′) was a meaningful amino acid in the germline-maintained positions. Meaningful amino acids were again defined as in the known exon analysis described above. In these positions (marked in red in Sup. Fig. 4), the abundance of GS′′N and S′PA were compared, normalized by the number of ″unique″ instances of amino acids at each position within the CDRs and the FWRs. Only 26 of the 27 positions had substitutions and therefore the comparison of substituted positions was only done on these 26 positions. The comparisons were done by comparing the medians of GS′′N and S′PA distributions across positions in the CDRs and FWRs using the Wilcoxon Signed Rank test. Individual A12 was not present in this analysis of the somatically substituted BCR sequence positions as we did not observe any substitutions in our 27 positions of interest in the BCR sequences of that individual.

### Analysis of phosphorylation sites from ExAC database

The Exome Aggregation Consortium (http://ftp.broadinstitute.org/pub/ExAC_release/release0.3.1/) (ExAC) combines the genetic variation observed in 60,706 unrelated healthy individuals. All observed single nucleotide variations (SNVs) are recorded for each position in the genome, along with the frequency of chromosomes that display this SNVs, and its quality score. The dataset contains an average of 12.5 SNVs per 100 nt in the exome. The Genome Reference Consortium Human Build 37 (GRCh37, hg19) was used for building this VCF version. We only considered high quality SNVs that are inside a canonical CDS. Stop codons were not considered within the CDS. These filtrations left us with 89.7% of the original data. SNVs were translated to single codon variations with respect to their position in the genome, using the our Geneffect Variant Effect Predictor (https://github.com/nadavbra/geneffect). Geneffect also provided a convenient interface for extracting sequence feature annotations from UniProtKB^32^. Specifically, out of 1,386,569 serine residues in hg19, a total of 30,219 sites were reported as phosphoserines according to Sequence features from UniProtKB. Based on these data, a human specific codon substitution matrix, was constructed. Each matrix cell represents the observed frequency of the substitution of a row codon by its column codon. The substitution of the major to the minor alleles is considered. A normalization was performed by the number of appearances of each codon in the canonical CDSs. This produced a 61 × 61 substitution matrix, several of the matrix cells are unoccupied due to the request for multiple steps for amino acid substitution. The diagonal entries, which represent the probability for a non-substitution, completed each row sum to 1. As a reference for neutral mutations, we created a 4-degenerate matrix, which considers only mutations that appear in the third base of 4 degenerate codons (it comprises ~86.5% of all mutations). The degenerate matrix was then converted to a nucleotide resolution, translating into a 4 × 4 mutation matrix. This 4-degenerate nucleotide matrix was translated back to a codon substitution 4-degenerat matrix. This is the joint probability of the single nucleotide mutations assuming independence of all 3 positions within a codon. For phosphorylation test we included experimentally validated phosphoserine (13,092 sites), phosphothreonine (3,326) and phosphotyrosine (873 sites) based on the Sequence features from UniProtKB.

## Supplementary information

Supplementary materials
