# Supplementary material for: Serine substitutions are linked to codon usage and differ for variable and conserved protein regions
Source: Sci Rep. 2019 Nov 21;9:17238. doi: 10.1038/s41598-019-53452-3 (PMC6872785; doi:10.1038/s41598-019-53452-3)

Serine substitutions are linked to codon usage and differ for variable and conserved protein regions

Gregory W. Schwartz[1] Tair Shauli[2] Michal Linial[3] and Uri Hershberg[4,5,6,*]

1. Department of Pathology and Laboratory Medicine, Perelman School of Medicine at the University of Pennsylvania, ifthenelse2244@gmail.com
2. School of Computer Science and Engineering, The Hebrew University of Jerusalem, tair.shauli@mail.huji.ac.il
3. Department of Biological Chemistry, Institute of Life Sciences, The Hebrew University of Jerusalem, michall@mail.huji.ac.il
4. Drexel School of Biomedical Engineering, Science and Health Systems, Drexel University
5. Department of Microbiology and Immunology, Drexel College of Medicine, Drexel University
6. Department of Human Biology, Faculty of Science, University of Haifa

***Corresponding author**

Uri Hershberg orcid.org/0000-0002-6425-5980

Tel: +1-203-6064230 email - uh25@drexel.edu , uri@sci.huji.ac.il

Supplemental figures

Supplemental figure 1a: The BLOSUM generated from the UCSC Genome Browser Database. The heat map is colored from blue (<-10) to red (>10) with a midpoint gradient of white (0).

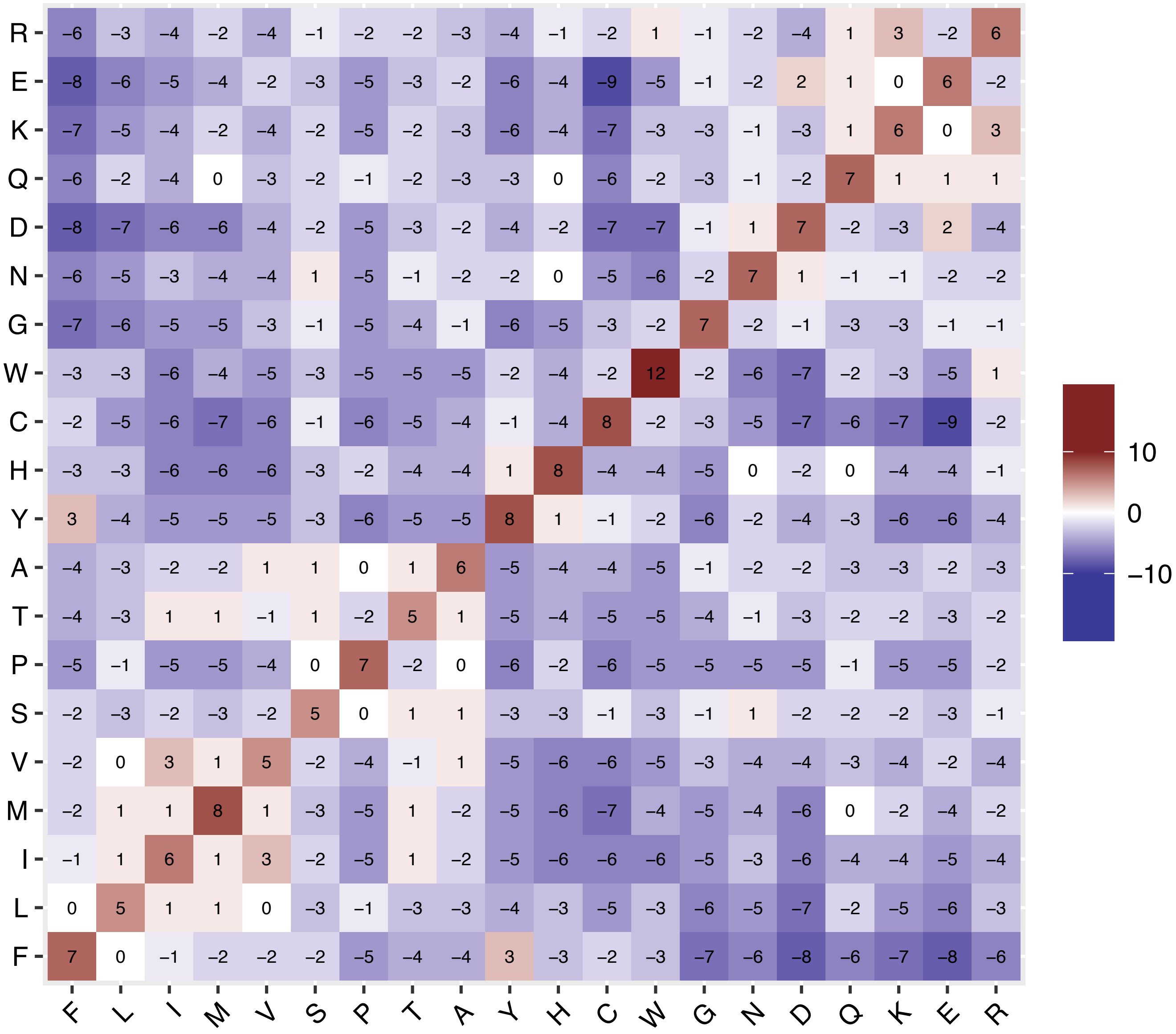

Supplemental figure 1b: The BLOSUM generated from the UCSC Genome Browser Database when arginine (R) is divided into 4fold synonymous and 2 fold synonymous codon set. The heat map is colored from blue (<-10) to red (>10) with a midpoint gradient of white (0).

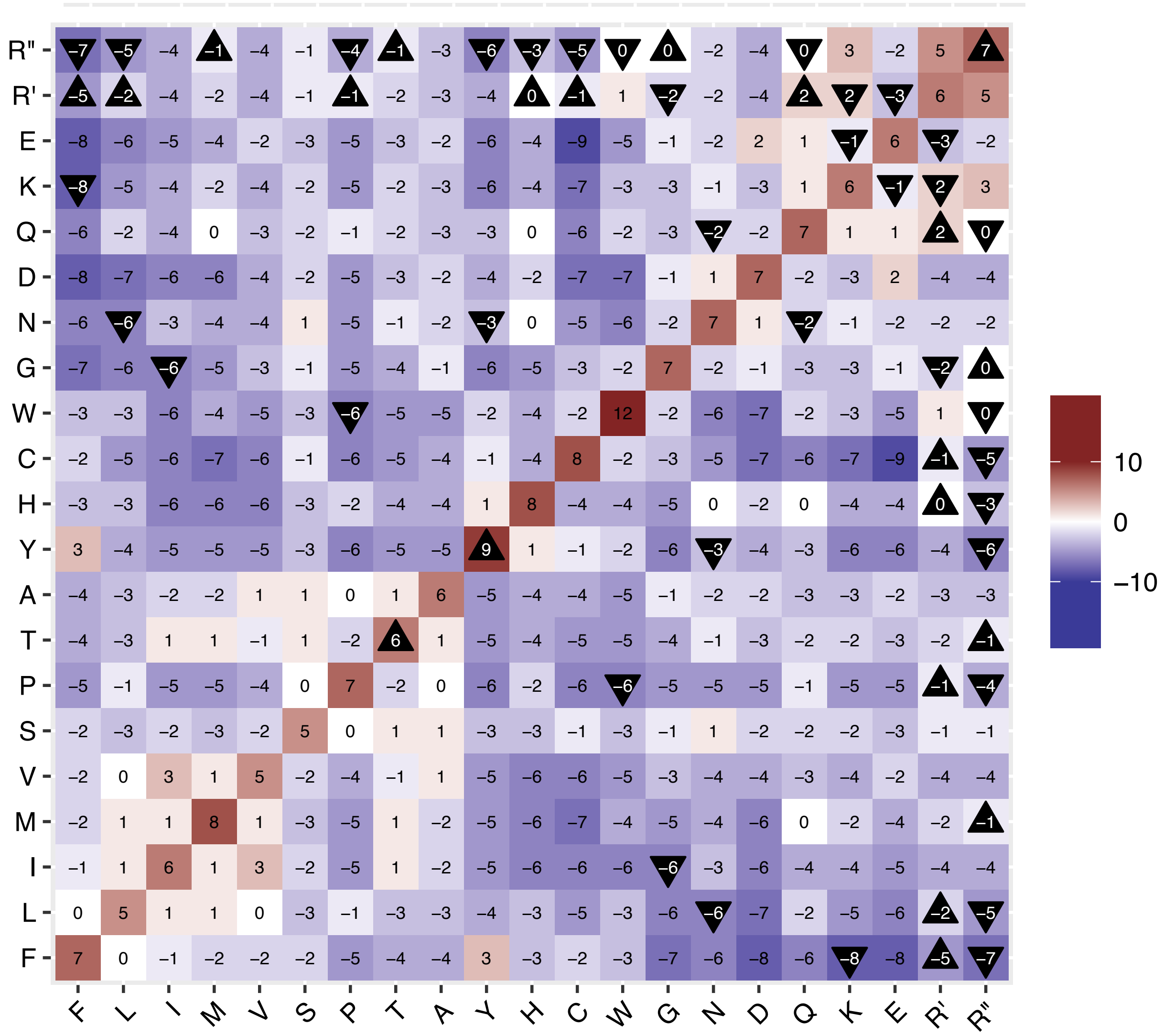

Supplemental figure 2: The amino acid makeup of gp120 in the HIV env gene at positions containing meaningful serines. The x axis shows the hypervariable regions highlighted in red, while the y axis is the number of individual sequences with a given amino acid at each position normalized by the height of the position which has the most amino acids at a position. Colored by amino acid type (*left*). Colored by GS″ND, S′PTA, or Other (*right*).

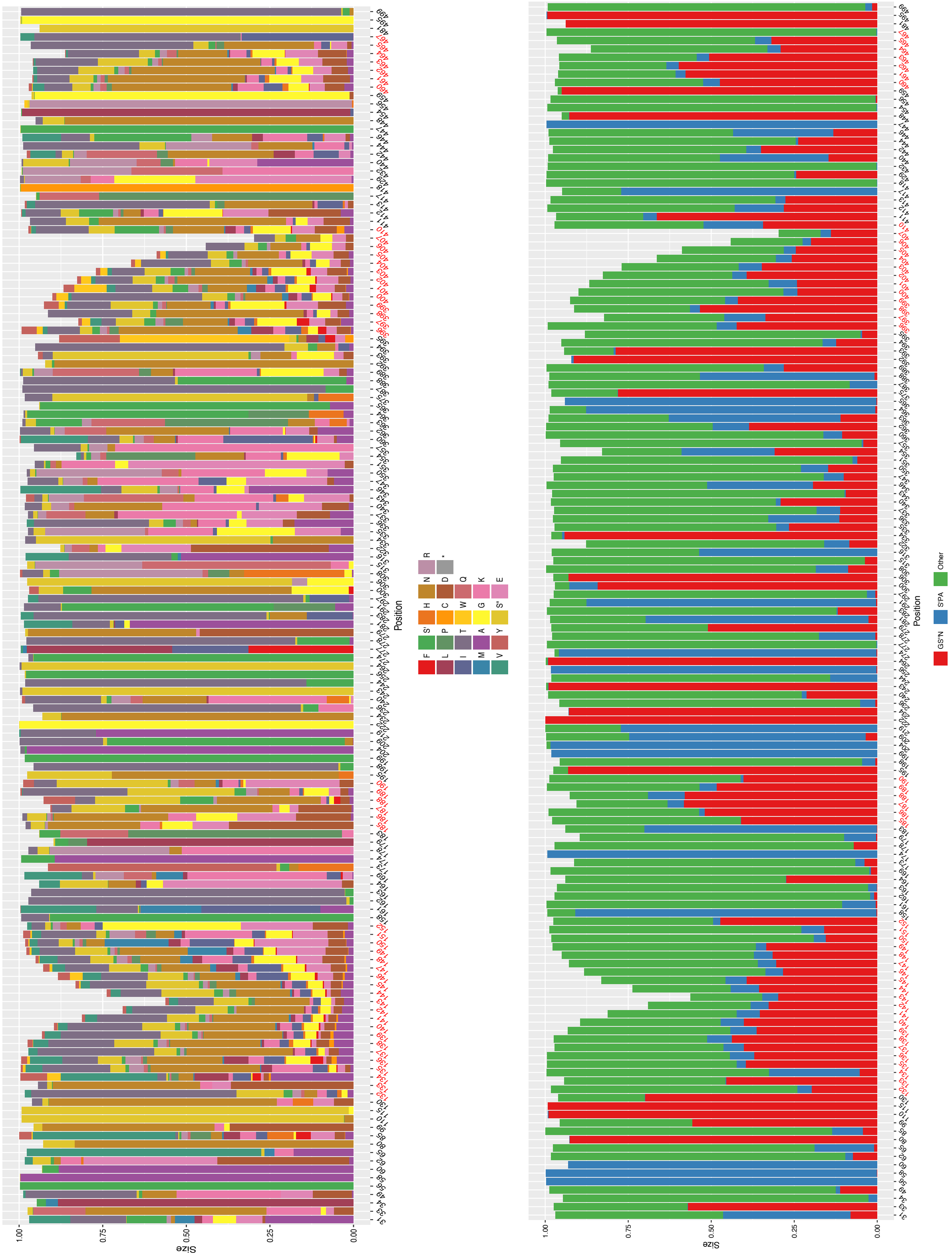

Supplemental figure 3: The amino acid makeup of germline amino acids at positions containing meaningful serines. The x axis shows FWR positions in black and CDR positions in red, while the y axis is the number clones with given amino acids at each position normalized by the height of the position which has the most amino acids at a position. Germline receptor positions colored by amino acid type (*top*). Germline receptor positions colored by GS$''$ND, S$'$PTA, or Other (*bottom*):

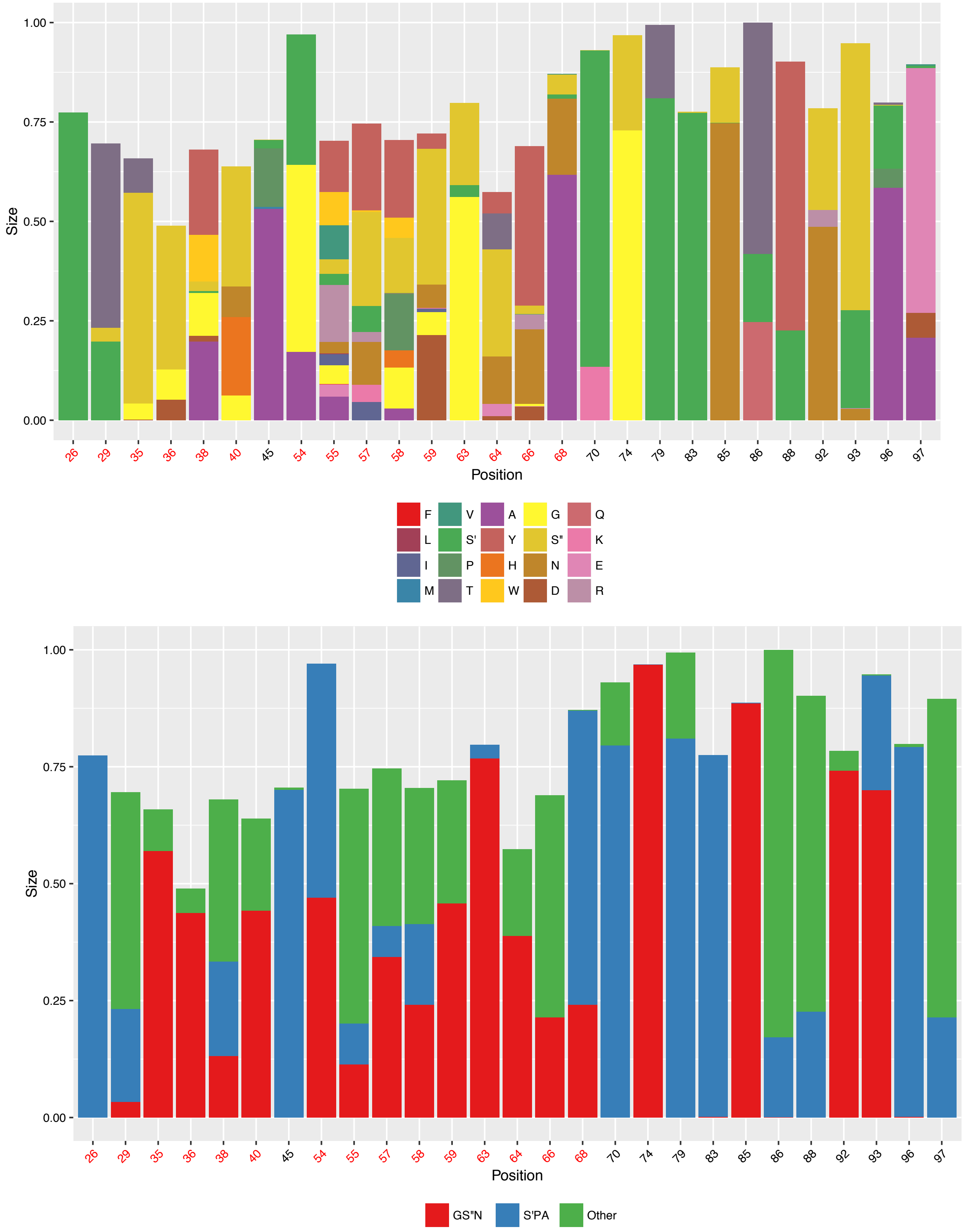

Supplemental figure 4: The amino acid makeup of somatic amino acids at positions containing meaningful serine residues. Somatic receptor positions colored by amino acid type

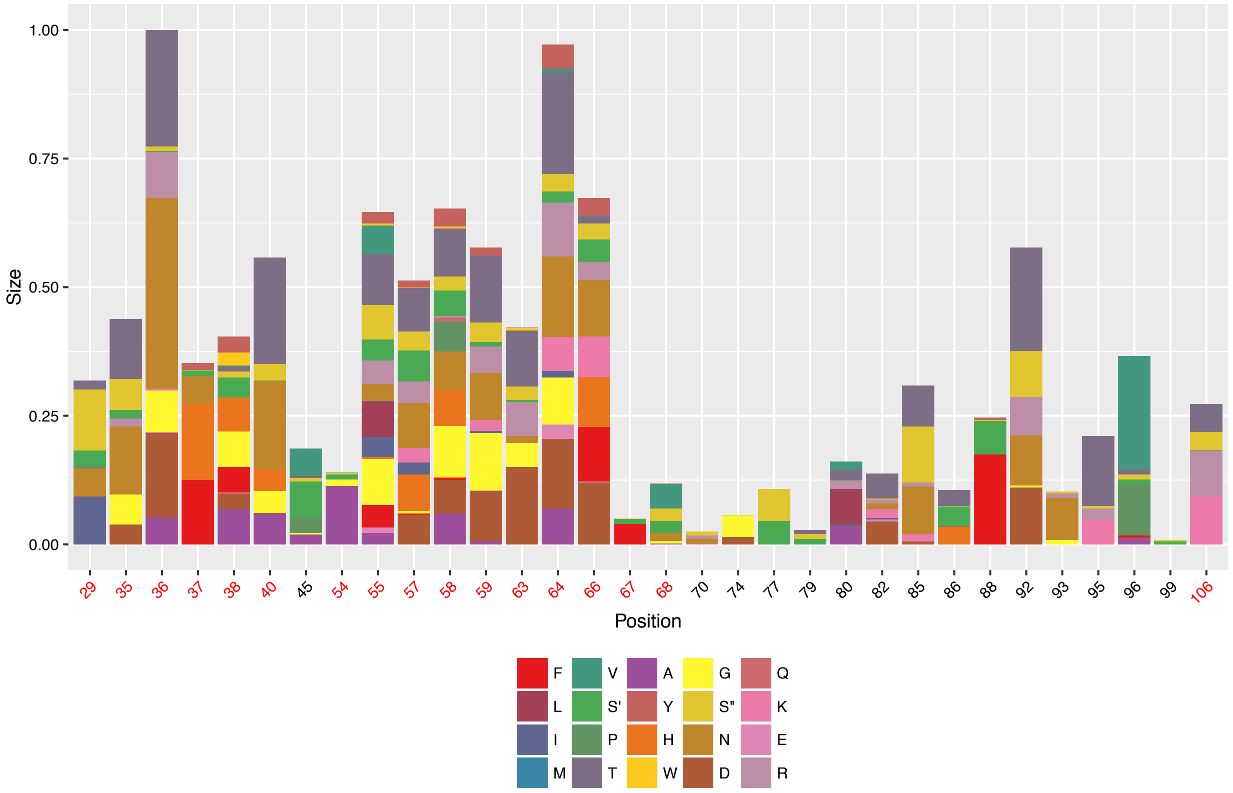

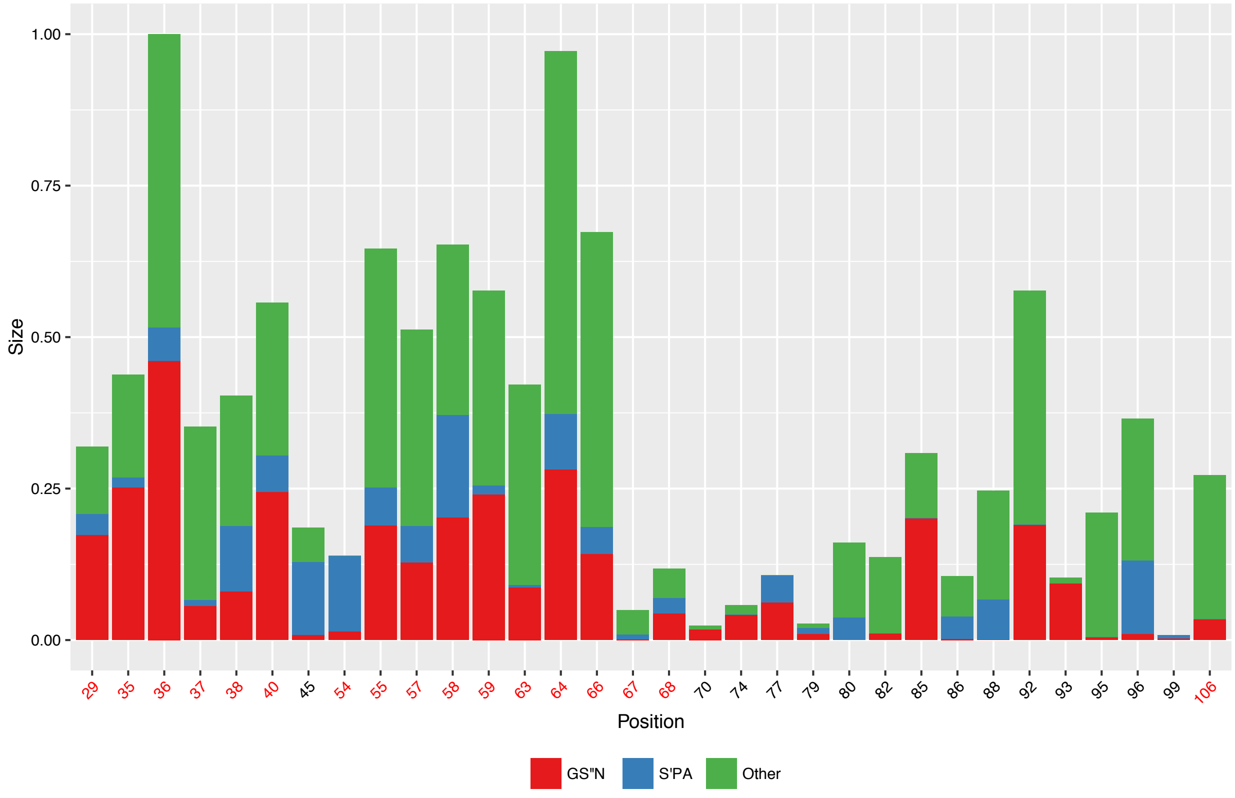

Supplemental figure 5: Mutation rates based on all polymorphisms observed at the third positions of the 4-fold degenerate amino acids according to aggregated data from the ExAC database.

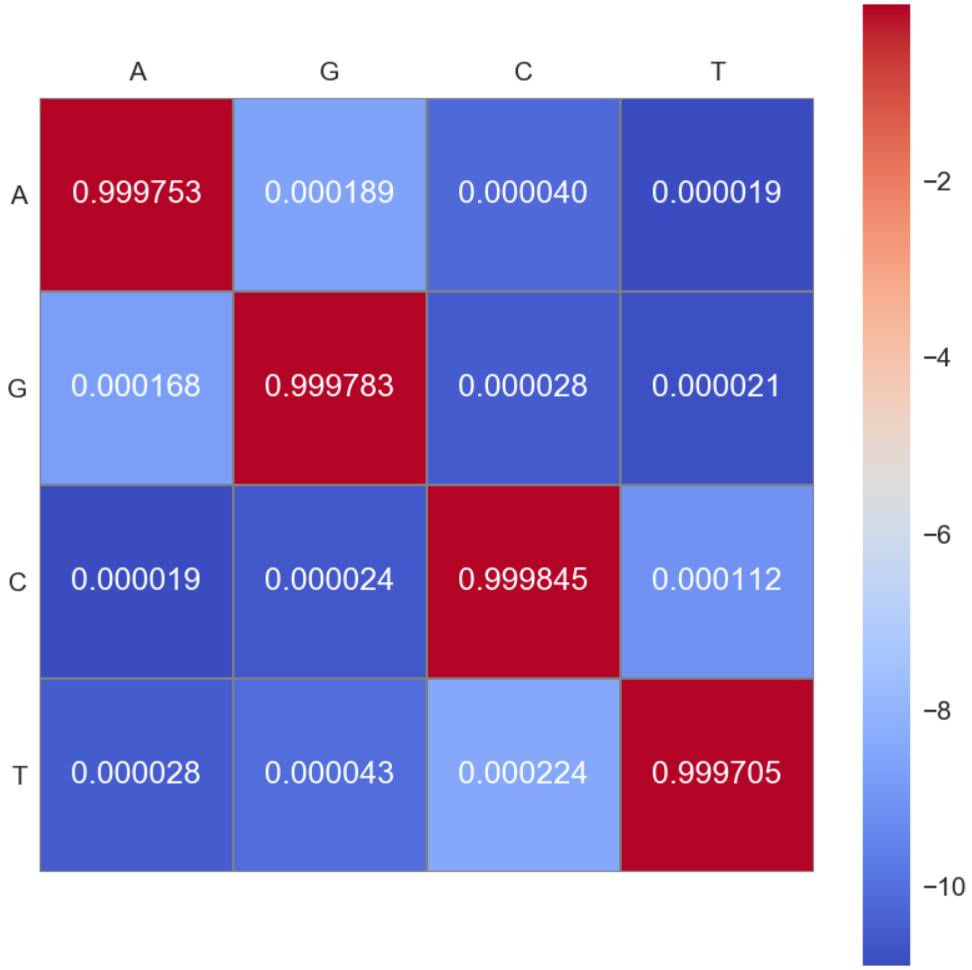

Supplement: Supplementary file 1 — Supplementary materials [file 41598_2019_53452_MOESM1_ESM.pdf]
